# Early Ambulation Shortened the Length of Hospital Stay in ICU Patients after Abdominal Surgery

**DOI:** 10.3390/clinpract13060141

**Published:** 2023-12-18

**Authors:** Takashi Amari, Daiki Matta, Yukiho Makita, Kyosuke Fukuda, Hiroki Miyasaka, Masami Kimura, Yuta Sakamoto, Satoshi Shimo, Kenichiro Yamaguchi

**Affiliations:** 1 Department of Rehabilitation, Health Science University, 7187 Kodachi, Fujikawaguchiko, Yamanashi 401-0380, Japan; kyosuke.fukuda@kenkoudai.ac.jp (K.F.); y.sakamoto@kenkoudai.ac.jp (Y.S.); sshimo@kenkoudai.ac.jp (S.S.); 2 Department of Anatomy and Life Structure, Juntendo University Graduate School of Medicine, 2-1-1 Hongo, Bunkyo-ku, Tokyo 113-8421, Japan; 3 Department of Rehabilitation, Ageo Central General Hospital, 1-10-10 Kashiwaza, Ageo, Saitama 362-8588, Japan; daiking3489@icloud.com (D.M.); df.mkhy@gmail.com (Y.M.); nv_k_row@icloud.com (H.M.); kimura.mas@ach.or.jp (M.K.); 4 Graduate School of Health and Sciences, Kyorin University, 5-4-1 Shimorenjaku, Mitaka-shi, Tokyo 181-8612, Japan; 5 Department of Rehabilitation, Sainokuni Higashi Omiya Medical Center, 1522 Toro-cho, Kita-ku, Saitama-shi 331-8577, Japan; kenichiro.yamaguchi@shmc.jp

**Keywords:** ambulation, intensive care unit, length of stay, patient discharge, postoperative complications

## Abstract

The optimal time to ambulation remains unclear for intensive care unit (ICU) patients following abdominal surgery. While previous studies have explored various mobilization techniques, a direct comparison between ambulation and other early mobilization methods is lacking. Additionally, the impact of time to ambulation on complications and disuse syndrome prevention requires further investigation. This study aimed to identify the optimal time to ambulation for ICU patients after abdominal surgery and considered its potential influence on complications and disuse syndrome. We examined the relationship between time to ambulation and hospital length of stay (LOS). Patients were categorized into the nondelayed (discharge within the protocol time) and delayed (discharge later than expected) groups. Data regarding preoperative functioning, postoperative complications, and time to discharge were retrospectively collected and analyzed. Of the 274 postsurgical patients managed in the ICU at our hospital between 2018 and 2020, 188 were included. Time to ambulation was a significant prognostic factor for both groups, even after adjusting for operative time and complications. The area under the curve was 0.72, and the cutoff value for time to ambulation was 22 h (sensitivity, 68%; specificity, 77%). A correlation between time to ambulation and complications was observed, with both impacting the hospital LOS (model 1: *p* < 0.01, r = 0.22; model 2: *p* < 0.01, r = 0.29). Specific cutoff values for time to ambulation will contribute to better surgical protocols.

## 1. Introduction

During the perioperative period of abdominal surgery, postoperative bed rest leads to intensive care unit (ICU)-acquired weakness (ICU-AW) and disuse syndrome [1], which not only prolong the hospital length of stay but also reduce patients’ quality of life (QOL) and increase medical costs. Early mobilization is a general activity that prevents disuse syndrome [2]. Specific techniques include standing from the bed, sitting up in bed, and walking. However, there is no consensus regarding which mobilization technique is most effective [3]. One study has shown that the length of time before walking independently (time to ambulation) is important to early mobilization, which can affect the hospital length of stay [4]. Therefore, it is necessary to consider ambulation as a mobilization technique that can prevent disuse syndrome.

Early ambulation is critical in intensive care physiotherapy because it significantly improves physical functions that have been reduced by lying in bed and improves QOL after discharge. Early ambulation in the ICU minimizes the effects of ICU-AW [5], shortens the hospital length of stay, and reduces complications [6]. Similarly, enhanced recovery after surgery (ERAS) protocols recommend early mobilization to promote postoperative recovery [7] and shorter hospital stays [8] in the surgical ICU.

Therefore, early ambulation can effectively reduce the hospital length of stay for ICU patients with disuse syndrome. However, the optimal time to ambulation after surgery for ICU patients has not been fully investigated. Recently, because the number of elderly patients undergoing surgery has increased, the number of patients who have been admitted to the ICU after abdominal surgery has increased [9,10,11]. Therefore, early ambulation in the ICU should be considered after abdominal surgery. Furthermore, the importance of early mobilization after abdominal surgery has been suggested in recent years, and it is actively being considered for ERAS protocols [3].

A study of patients who underwent minimally invasive surgery for gastric cancer found that respiratory function and time to ambulatory independence were factors that affect the hospital length of stay [4]. In the case of surgery, such as pancreaticoduodenectomy and hepatectomy, early ambulation on postsurgical day 1 is recommended; however, the level of evidence is low [12]. Compared to minimally invasive surgeries, highly invasive surgeries place a heavier burden on the body. Therefore, postoperative vital signs are more labile, resulting in delayed ambulation. Additionally, complications may prolong the hospital length of stay. Regarding abdominal surgery, examining the relationship between the specific time to ambulation and hospital length of stay may provide a specific indicator of when patients should start ambulating in the ICU, thus possibly contributing to shorter hospital stays.

This study aimed to determine the relationship between the time to ambulation initiation and other factors that prolong the hospital length of stay of patients admitted to the ICU after abdominal surgery and investigate specific time to ambulation cutoff values for prolonged lengths of stay.

## 2. Materials and Methods

This retrospective study investigated the causal relationship between time to ambulation initiation and hospital length of stay. Patients included in the study were divided into two groups. The nondelayed group included those who were discharged within the number of days stated in the hospital disease protocol, and the delayed group included those who were discharged later than expected according to the hospital disease protocol.

**Figure 1:**
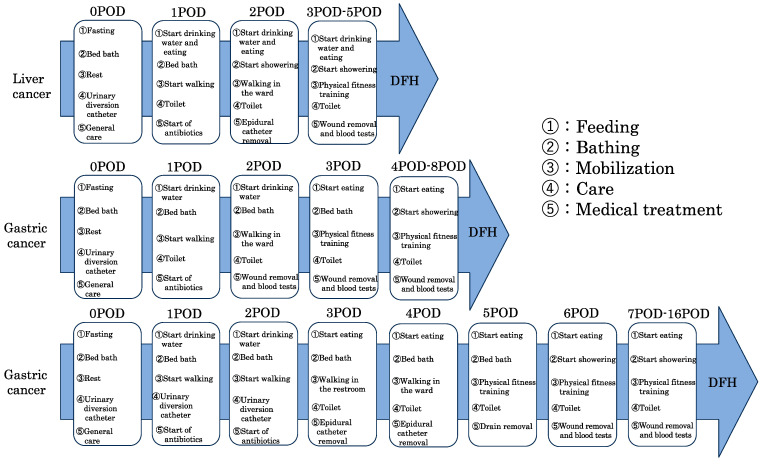
Clinical paths for typical diseases. DFH; discharge from hospital.

The inclusion criteria were gastrointestinal surgery, admission to the ICU, and rehabilitation between 2018 and 2020. This study was conducted at a large hospital that can handle major surgeries and employs many physical therapists dedicated to the ICU. Additionally, well-trained staff members are available for patient interventions. Perioperative protocols for typical diseases are shown in Figure 1. The protocols are shared by the ICU team, and treatment is performed according to these protocols. Mobilization is handled by physical therapists.

For surgeries without surgical protocols, patients who stayed in the hospital longer than the planned discharge date, which was set by the attending physician during the preoperative explanation to the patient, were classified as the delayed group.

The exclusion criteria comprised emergency surgery, death, or hospital transfer. During this study, we excluded 77 patients who underwent emergency surgery, 7 patients who died, and 2 patients who were transferred to another hospital. The remaining 188 patients were included in the analysis. All these patients had no cognitive impairment before admission and were able to walk independently during their daily lives. The following data were retrospectively collected: age, sex, height, surgical procedure, preoperative vital capacity (%VC), in-hospital complications, postoperative hospital length of stay, operative time, total blood loss, time to ambulation, reasons for nonambulation, time to standing from the hospital bed, and Acute Physiologic Assessment and Chronic Health Evaluation (APACHE) II score. The postoperative hospital length of stay was from the day of surgery (day 0) to the day of discharge. Postoperative complications were defined as complications that required treatment. Preoperative vital capacity (%VC) is a pulmonary function used to assess the risk of complications before surgery during the perioperative period [4]. Previous studies have reported that %VC is associated with the hospital length of stay. According to previous studies, operative time and total blood loss also affect the hospital length of stay [13]. To further assess the weaning process, weaning data including the time to ambulation, time to wheelchair sitting, time to end of wheelchair dependence, and time to standing from the hospital bed were collected. Disease severity was assessed using the APACHE II score, which is the most commonly used scoring system for determining the severity of illness worldwide [14].

Statistical analyses were performed using JMP version 11.5 (SAS Institute Inc., Cary, NC, USA) and SPSS Amos (IBM Corp., Armonk, NY, USA). We compared the nondelayed and delayed groups according to differences in early discharge delays. The dependent variable for both the delayed and nondelayed groups was the postoperative hospital length of stay. This study was conducted at Ageo Central General Hospital in Japan between 2018 and 2020. Data regarding time to first ambulation, time to wheelchair sitting, time to end of sitting, and time to standing from the hospital bed were collected during a 4-month period from April to August 2020, and examined using the unpaired t-test or chi-squared test to determine each group’s attributes. During the analysis of factors associated with the hospital length of stay, the hospital length of stay was a binary variable (delayed group vs. nondelayed group). Therefore, we performed a logistic regression analysis.

The logistic regression analysis was performed using the forced imputation method, with the hospital length of stay (in days) as the dependent variable, time to ambulation as the exposure, ambulation items as covariates, and preoperative items and ambulation-related items as confounders. To check for multicollinearity of the input items, a regression analysis was performed. Items with a regression coefficient ≥0.9 were excluded. Additionally, the goodness-of-fit of the model in the logistic regression analysis was determined using the Akaike information criterion (AIC). We conducted a path analysis for each factor to confirm the relationship between factors. A path analysis has validity in the medical field because it allows for the examination of inter-relationships of factors [15,16]. We created models using the obtained factors and then evaluated the validity of those models. The fit of the model in the path analysis was evaluated using a comparative fit index (CFI), AIC, and a root mean square error of approximation (RMSEA). In general, a CFI close to 1 and an RMSEA less than 0.07 are considered to indicate a good fit for the model [17].

This study was conducted with the approval of the Ethics Committee of Ageo Central General Hospital (approval no. 829). The study was conducted with the written consent of all research participants.

## 3. Results

**Figure 2:**
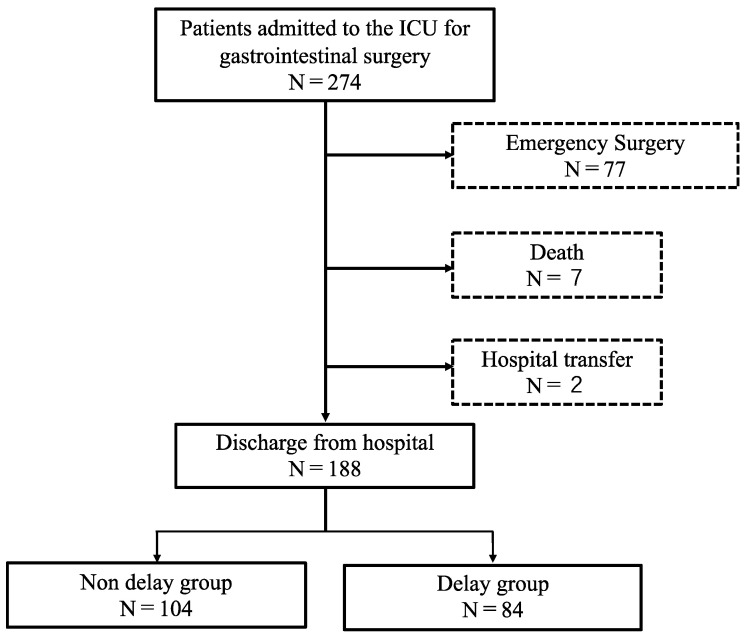
Patient selection for this study.

Of the 274 patients who underwent invasive abdominal surgery and subsequent ICU management during this period, 188 patients who underwent surgery and rehabilitation were included in the analysis (Figure 2). During this study, 73% of patients underwent bile duct pancreatic surgery. The proportions of patients who underwent upper and lower digestive tract surgeries were similar (Table 1).

**Figure 3:**
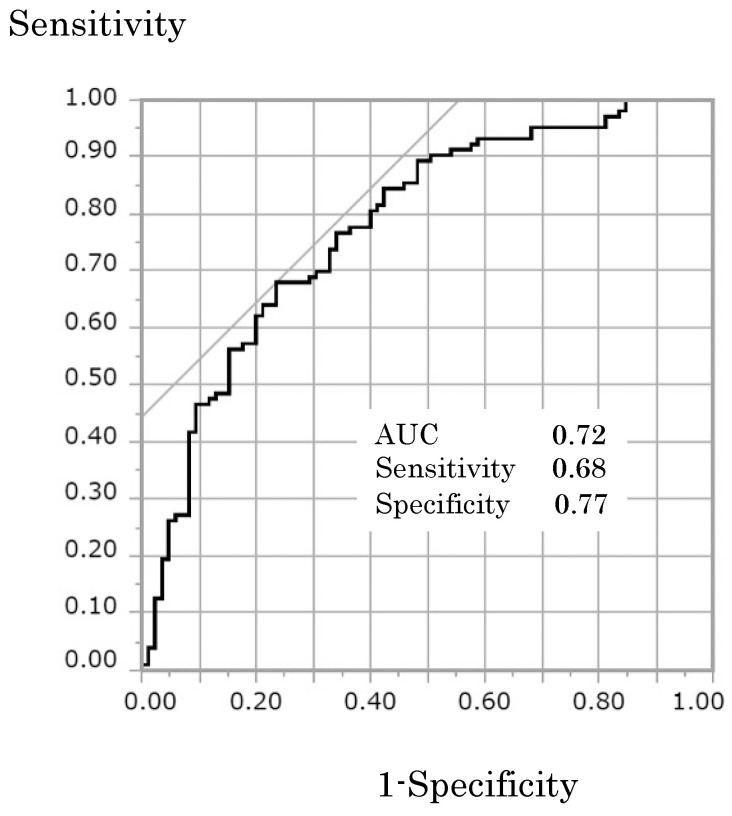
Receiver-operating characteristic (ROC) curve associated with the cutoff time calculation for time to ambulation initiation related to the hospital length of stay. AUC is the area under the curve.

A comparison between the non-delayed and delayed groups showed that the delayed group had significantly longer operative times, greater total blood loss, more complications, and a longer time to ambulation (Table 2). The results of the logistic regression analysis depicted in Table 3 show that for both the delayed and non-delayed postoperative hospital stay groups, the time to ambulation (*p* < 0.01; odds ratio [OR], 0.99; 95% confidence interval [CI], 0.993–0.999) was a significant variable. The other significant factors were the presence of complications (*p* < 0.01; OR, 0.14; 95% CI, 0.054–0.345) and operative time (*p* < 0.05; OR, 0.99; 95% CI, 0.999–0.994). In Figure 3, the receiver operating characteristic (ROC) curve showed that the area under the curve (AUC) was 0.77; the cutoff value for time to ambulation was 22 h. The sensitivity and specificity of this cutoff value were 68% and 77%, respectively. The high specificity suggests that time to ambulation, together with other factors, may predict delayed hospital discharge.

**Figure 4:**
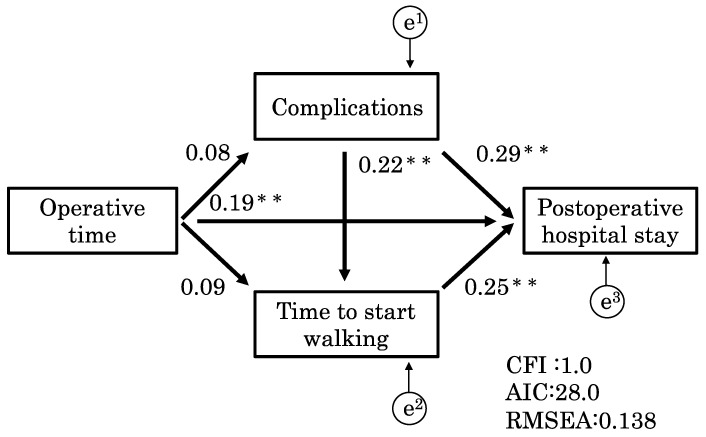
Model 1. This model shows how complications affect time to ambulation. Model 1 had a CFI of 1.0, an AIC of 28.0, and an RMSEA of 0.14. The models demonstrated that the time to ambulation and complications (*p* < 0.01; r = 0.22) interacted with each other and affected the hospital length of stay. CFI, comparative fit index; AIC, Akaike information criterion; RMESEA, root mean square error of approximation. ** *p* < 0.01.

**Figure 5:**
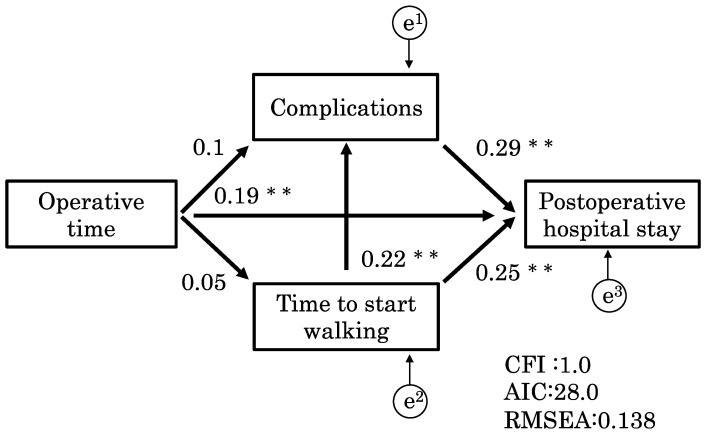
Model 2. This model shows how time to ambulation affects complications. Model 2 had a CFI of 1.0, AIC of 28.0, and RMSEA of 0.14. Model 2 was more effective and had a stronger effect on the hospital length of stay than model 1 (*p* < 0.01; r = 0.29). CFI, comparative fit index; AIC, Akaike information criterion; RMESEA, root mean square error of approximation. ** *p* < 0.01.

We investigated the relationship between factors related to the hospital length of stay using a path analysis. Operative time (r = 0.24; *p* < 0.01), complications (r = 0.43; *p* < 0.01), and time to ambulation (r = 0.27; *p* < 0.01) were related to the hospital length of stay, whereas complications were related to the time to ambulation (r = 0.17; *p* < 0.01) (Table 4). These results were significant for indicating an association, but the strength of the association was mild. A path analysis was performed to create two models. Model 1 (Figure 4) had a CFI of 1.0, AIC of 28.0, and RMSEA of 0.14. Similarly, model 2 (Figure 5) had a CFI of 1.0, AIC of 28.0, and RMSEA of 0.14. These models did not have significantly different fit, with a CFI of 1.0 indicating a good fit and an RMSEA of 0.14 indicating a fair fit.

The models demonstrated that the time to ambulation and complications (*p* < 0.01; r = 0.22) corresponded and affected the hospital length of stay. Additionally, model 2 was more effective and had a stronger effect on the hospital length of stay than model 1 (*p* < 0.01; r = 0.29). This suggests that these two models had the same fit, but that the path from time to ambulation to the hospital length of stay via complications was more strongly associated in model 2 than in model 1.

## 4. Discussion

The time to ambulation and its effect on the hospital length of stay for patients who underwent abdominal surgery and were admitted to the ICU were investigated. However, whether the process of initiating postsurgical ambulation affects the hospital length of stay or other factors during the course of hospitalization remains unclear. Although the ERAS protocols recommend that patients should start walking on the first postsurgical day after pancreaticoduodenal surgery, the degree to which varying times to ambulation affect outcomes has not yet been studied [18].

This study is clinically significant because it enabled us to examine the effect that time to ambulation has on the hospital length of stay and further allowed for more focus on initiating postsurgical ambulation in the ICU.

We hypothesized that the time to ambulation affects the hospital length of stay. A logistic regression analysis, including confounding, showed that the time to ambulation, even after adjusting for operative time and complications, had a crucial role in determining the hospital length of stay. Previous studies have reported that operative time and complications can prolong the hospital length of stay [19,20], and our results support those results. We demonstrated for the first time that not only the operative time and complications but also the time to ambulation are related to the hospital length of stay. The association between delayed time to ambulation (>1 day) and hospital length of stay has been shown previously [4,21]. Some previous studies have reported that comprehensive interventions, including walking, prevent complications and reduce the number of days in the hospital [22,23]. This is the first study to examine the cutoff of delayed ambulation for patients who have undergone abdominal surgery. Based on the ROC curve, the cutoff value for time to ambulation was 22 h.

Ni et al. reported that early mobilization (walking within 2 days of surgery) was associated with shorter hospital length of stay and improved gastrointestinal function of patients who underwent surgery for liver cancer, but there was no significant difference in complications [24]. However, other studies reported that early mobilization on the day of surgery for patients with pancreatic cancer can improve oxygenation in the short term [25] and that early mobilization may prevent postoperative complications. Based on these reports, the association between early mobilization and complications has not yet been definitively established.

The reason why this study defined early mobilization as walking is because many previous studies have not distinguished walking from sitting up in bed. During this study, we focused on walking for early mobilization. In particular, the cutoff time to ambulation of 22 h in this study referred to walking and was more exact than the goal of leaving the bed within 24 h used by previous studies. In clinical practice, it may be necessary to consider, for example, starting walking in the evening if surgery is completed in the morning, and starting walking in the morning or afternoon if surgery is completed in the evening. This suggests that early initiation of ambulation, as proposed by the ERAS protocol, can shorten the hospital length of stay for patients who have undergone abdominal surgery. Although the participants in this study were able to independently perform activities of daily living, their preoperative physical function [4] may have influenced the initiation of ambulation; however, the preoperative physical function was not considered during this study.

Operative time and complications affect the transfer destination of patients. Previous studies have shown that delayed postoperative ambulation after minimally invasive surgery increases the risk of complications [21,26,27]. Therefore, during this study, we performed a path analysis to examine factors that affect the hospital length of stay after invasive surgery. Significantly, these factors were sequentially quantified by the path analysis during this study. The results of models 1 (Figure 4) and 2 (Figure 5) indicated that, along with complications and operative time, postsurgical time to ambulation affects the hospital length of stay; however, complications and time to ambulation were related factors that affected hospital stay. Furthermore, this study showed that the time to ambulation and complications correspond to each other, suggesting that complications may affect the time to ambulation and increase the hospital length of stay.

Models 1 and 2 showed that the pathway from time to ambulation to hospital length of stay via complications was more significant than the pathway from complications to hospital length of stay via time to ambulation. This means that there is a series of pathways in which the time to ambulation affects the hospital length of stay attributable to complications. These results suggest that, in clinical practice, it is critical to focus attention on complications originating from delayed postsurgical ambulation to prevent a prolonged hospital stay.

Rivas et al. reported that the bed rest duration, including sitting and standing after surgery, was significantly associated with complications, with pneumonia being the most common postoperative complication [28]. The most common postoperative complication during this study was pancreatic fistula, followed by pneumonia, which is consistent with previous studies. This study included many pancreatic and liver surgeries, with an average operative time of 6 to 7 h, which means that patients were in the supine position during surgery for long periods. Anesthesia, muscle relaxants, and opioids used for postoperative pain management can decrease the cough reflex and accessory muscle function [22]. Additionally, staying in the same position for a long time can lead to decreased ventilation and difficulty expectorating sputum, thus increasing the risk of pneumonia [4]. Furthermore, abdominal surgery can cause abdominal edema, which can increase intra-abdominal pressure and impair ventilation function, even in the sitting position. Although the average preoperative %VC in this study was more than 100%, the increased risk of pulmonary complications may have been associated with the combination of long operative times and delayed walking. On the other hand, studies have shown that early mobilization compliance is quite low among patients who have undergone colorectal surgery. Specifically, it has been reported that less than 50% of patients left the bed on the first day after surgery [29]. During this study, the delayed group started sitting after approximately 25 h, and the average time to ambulation was 52 h. However, further investigation is necessary to understand the reasons for the lack of mobilization.

The results of this study provided time-based numerical values and specific mobilization methods that will contribute to the establishment of more detailed protocols. Additionally, sharing these results with various healthcare professionals and patients in the ICU will lead to beneficial outcomes. For example, by coordinating postoperative schedules to initiate walking as soon as possible, complications may be prevented. Additionally, explaining the importance of early walking to patients may increase their cooperation, and discharge can occur as planned.

This study was limited because we did not sufficiently examine each specific disease. For example, surgical invasiveness and postoperative complications differ between the lower gastrointestinal tract and pancreas. Therefore, disease-specific applications of this research are necessary. Future studies should consider separating various classes of diseases and examining them independently. Furthermore, because the time to ambulation was calculated from the time when surgery ended, the time to ambulation may have been delayed by factors related to the medical staff. For example, a patient whose surgery was completed during the day will have a longer time to ambulation than that of a patient whose surgery was completed at night. Therefore, future studies should include the time constraints of the medical staff.

## 5. Conclusions

We examined factors that influence the hospital length of stay after invasive abdominal surgery and found that time to ambulation was a significant factor when the cutoff value was 22 h. Further examination of the relationship between these factors revealed that although time to ambulation was associated with complications, it had a stronger effect on the hospital length of stay.

## Figures and Tables

**Table 1:** Number of participants by surgery.

Surgical Site	Surgery	Participants, No.	Hospital Length of Stay, Days (Quartile Range)
Upper digestive tube surgery (N = 23) 12%	Laparoscopic gastrectomy	11	14 (10.8–30.5)
	Thoracoscopic laparoscopic esophageal extraction	10	20.5 (16.3–26.3)
	Open gastrectomy	2	11
Lower digestive tube surgery (N = 28) 15%	Laparoscopic rectal resection	9	21 (11–41.5)
	Laparoscopic S-shaped colectomy	9	16 (10–31)
	Laparoscopic colectomy	4	18 (7.5–32)
	Laparoscopic ileal resection	3	6
	Open rectal resection	2	15.5 (11–20)
	Open colectomy	1	21
Bile duct pancreas surgery (N = 137) 73%	Laparoscopic hepatectomy	78	8.5 (7–13.3)
	Pancreatic head and duodenectomy	47	22 (14.8–42.3)
	Caudal pancreatectomy	5	44 (32–65)
	Central pancreatectomy	2	28.5 (10–47)
	Other choledochopancreatic surgery	5	23 (11–59)

Laparoscopic hepatectomy and pancreatic head and duodenectomy surgeries accounted for the largest proportions of surgeries.

**Table 2:** Basic characteristics of the participants.

Characteristics	Nondelayed Group	Delayed Group	*p*-Value
Age	70.2 ± 0.9	72.8 ± 1.1	0.07
Diagnosis			
Liver cancer	60	17	0.01 **
Pancreatic cancer	7	27	
Gastric cancer	11	6	
Colorectal cancer	4	8	
Esophageal cancer	4	6	
Bile duct cancer	6	9	
Other	12	9	
Tumor stage			
0	4	2	0.15
Ⅰ	0	0	
Ⅱ	15	7	
Ⅲ	78	66	
Ⅳ	7	9	
BMI (%) ^a^	22.9 ± 0.3	22.1 ± 0.4	0.12
Operative time (min)	362.3 ± 16.2	434.6 ± 17.6	0.01 **
Amount of blood loss (mL)	331.6 ± 72.4	575.3 ± 78.6	0.02 *
Complications (N)	7	34	0.01 **
Delirium	3	3	–
Intestinal obstruction	1	4	
Atelectasis	2	4	
Pneumonia	1	8	
Decreased appetite caused by decreased activity	0	1	
Pancreatic fistula	0	11	
Rupture of false aneurysm	0	3	
APACHE II score ^b^	10.3 ± 0.2	10.9 ± 0.5	0.24
%VC ^c^	103.6 ± 2.1	100.4 ± 2.2	0.29
Postoperative hospital stay	9.53 ± 2.29	39.9 ± 2.5	0.01 **
Complications (yes/no)	7/96	18/67	0.01 **
Time to ambulation (min)	2100.6 ± 224.9	3132.4 ± 247.1	0.01 **
Time to wheelchair sitting (min)	1704.9 ± 108.9	1943.6 ± 120.4	0.14
Time to sitting upright (min)	1343.6 ± 75.1	1511.3 ± 82.9	0.14
Time to standing from bed (min)	1083.9 ± 26.3	1055.1 ± 29.2	0.64

Data are presented as mean ± SD unless otherwise indicated. * *p* < 0.05; ** *p* < 0.01. ^a^ BMI: body mass index; ^b^ APACHE II score: Acute Physiology and Chronic Health Evaluation 2 score; ^c^ %VC: vital capacity percentage. Time to first ambulation, time to wheelchair sitting, time to end of wheelchair sitting, and time to standing from the hospital bed were examined using the unpaired t-test or chi-square test to determine each group’s attributes. Significant differences were observed in the operative time, amount of blood loss, postoperative hospital length of stay, complications, and time to ambulation.

**Table 3:** Results of the logistic regression analysis of the hospital length of stay.

	*p*-Value	Odds Ratio	95% CI	
			Upper Limit	Lower Limit
%VC	0.17	1.01	1.023	0.998
Time to sitting upright	0.94	1.00	1.010	0.999
Time to ambulation	0.01 **	0.99	0.993	0.999
Complications	0.01 **	0.14	0.345	0.054
Operative time	0.05 *	0.99	0.999	0.994

* *p* < 0.05; ** *p* < 0.01. The logistic regression analysis was performed using the forced imputation method, with the number of days in the hospital as the dependent variable, time to ambulation as the exposure, bed release items as covariates, and preoperative items and bed release-related items as confounders.

**Table 4:** Correlations of each factor.

	Postoperative Hospital Stay	Operative Time	Complication	Time to Ambulation
Postoperative hospital stay (days)	-	0.24 **	0.43 **	0.27 **
Operative time (min)	0.24 **	-	0.12	0.07
Complications	0.43 **	0.12	-	0.17 **

** *p* < 0.01. A regression analysis was performed to confirm the relationship and multicollinearity of each factor. Items with regression coefficients >0.9 were excluded.

## Data Availability

The data generated or analyzed during the current study are available from the corresponding author upon reasonable request.
